# A New Dental College

**Published:** 1867-08

**Authors:** 


### A NEW DENTAL COLLEGE.

A Dental College or Institute, we learn has recently been organized, in connection with, or rather under the auspices of Harvard University.

From the proposed arrangements we have little doubt it will be eminently successful; it is under the fostering care and guidance of an old and well established institution. A part of the teachers are to be selected from the members of the medical faculty, and the members of the faculty from the Dental profession are required also to be M. D's.

The Dental course is to be given at a different time from that of the medical, so that a very strong objection that has been urged against alliances of this kind, is in this case removed.

The session, we believe, is to be held during the spring and summer, which in that latitude would be quite practicable, but in some others would be somewhat objectionable.

We suggested years ago, that New England should have its Dental College, and we feel well assured that this Institution will meet the demand. We shall take pleasure in announcing the names of the incumbents of the various chairs, as soon as they are made known.

